# A Case of Fournier's Gangrene Following the Initiation of Dapagliflozin

**DOI:** 10.7759/cureus.63168

**Published:** 2024-06-25

**Authors:** Natalie Shaykh, Avni Agrawal, Melville C O'Brien, Oshin Rai, Vanshika Tripathi, Vishal Jaikaransingh

**Affiliations:** 1 Internal Medicine, University of Florida College of Medicine – Jacksonville, Jacksonville, USA; 2 Nephrology, University of Florida College of Medicine – Jacksonville, Jacksonville, USA

**Keywords:** surgical intensive care unit (sicu), urology, scrotal pain, dapagliflozin, fournier's gangrene (fg)

## Abstract

Since being approved by the United States Food and Drug Administration (FDA) in 2013, sodium-glucose cotransporter-2 inhibitors (SGLT2is) have emerged as an appealing therapeutic choice for patients with diabetes due to their favorable effects on renal and cardiac health. Recent trials have further expanded the application of these drugs by showing a decrease in mortality rates among patients with both reduced and preserved ejection fraction heart failure, even in those without diabetes. Common adverse effects of SGLT2is include increased urinary frequency and urinary tract infections stemming from elevated glycosuria. Here, we present a case report involving a 66-year-old man who developed Fournier’s gangrene (FG) shortly after initiating dapagliflozin - a rare but dangerous adverse effect associated with this medication.

## Introduction

Sodium-glucose cotransporter-2 inhibitors (SGLT2is) represent a burgeoning drug category that can improve glycemic management and promote weight loss. Their mechanism entails decreasing plasma glucose levels by inhibiting glucose reabsorption in the kidney’s proximal tubules, increasing glycosuria [1]. Typical side effects encompass genital mycotic and urinary tract infections; however, after United States Food and Drug Administration (FDA) approval, additional adverse effects including acute kidney injury, ketoacidosis, urosepsis, pyelonephritis, and Fournier’s gangrene (FG) have surfaced [2,3]. FG is a necrotizing infection that impacts the soft tissues surrounding the perineum, perianal areas, and external genitalia, representing a urologic emergency [3]. Prior studies indicate that the occurrence of FG is approximately 1.6 cases per 100,000 in males and 0.25 cases per 100,000 in females within the United States [2,4,5]. The infection is typically polymicrobial with anaerobic bacteria being the predominant pathogens [5,6]. While the exact mechanism is unknown, it is hypothesized that the elevated glycosuria induced by SGLT2is fosters an environment for infection growth in the urinary and genital regions, potentially resulting in the onset of FG [7]. We present a case of FG that occurred following the initiation of dapagliflozin in a patient with predisposing factors for the development of FG. The goal of this case report is to inform clinicians of a rare side effect of SGLT2is, a medication that is now a staple in the management of heart failure, renal disease, and diabetes.

## Case presentation

This is the case of a 66-year-old male with a past medical history of hypertension, hyperlipidemia, type II diabetes mellitus (T2DM), coronary artery disease (CAD) status post coronary artery bypass graft, severe ischemic cardiomyopathy/heart failure with reduced ejection fraction of 15%-20% status post cardiac resynchronization therapy with defibrillator, left hip fracture status post open reduction and internal fixation, and deep vein thrombosis (DVT) on eliquis who was started on the SGLT2i dapagliflozin two months before his initial presentation. He presented to the emergency department (ED) after a fall at ground level, during which he remained on the floor for four days until discovered by his neighbor. He reported hitting his head and left hip. Alongside left hip pain, he complained of left scrotal pain and swelling, which worsened after the fall due to hip immobility. On arrival, his vital signs were normal, but he was soiled with dirt and urine. Physical examination revealed tenderness and swelling of the left testicle with normal cremasteric reflex, restricted left hip movement, healing wounds on the extremities without signs of infection, and a 2-cm stage 2 healing decubitus ulcer on the right buttock. Laboratory findings showed acute kidney injury, wide anion gap metabolic acidosis in the setting of lactic acidosis, hyperglycemia (333 mg/dL) with urinalysis indicating high glucose levels (>500 mg/dL), leukocytosis (28 thou/cumm), elevated creatinine kinase (376 U/L), and elevated troponins peaking at 123 ng/L, interpreted as secondary to acute on chronic myocardial injury by cardiology (Table 1).

**Table 1:** Initial laboratory findings on presentation

Complete Metabolic Panel	Value	Reference Range
Sodium	134	135-145 mmol/L
Potassium	3.9	3.3-4.6 mmol/L
Chloride	88	101-110 mmol/L
Carbon Dioxide	24	21-29 mmol/L
Urea Nitrogen	68	6-22 mg/dL
Creatinine	2.03	0.51-0.96 mg/dL
Blood Urea Nitrogen (BUN)/Creatinine Ratio	33.5	6-22
Estimated Glomerular Filtration Rate (eGFR)	35	≥ 60 mL/min/1.73M^2^
Glucose	333	71-99 mg/dL
Calcium	9.8	8.6-10.0 mg/dL
Osmolality Calculated	301	275 to 295 mOsm/kg
Creatinine kinase	376	< 200 U/L
Lactic Acid	3.7	0.7 - 2.7 mmol/L
Complete Blood Count and Differential		
White Blood Cell (WBC)	28.4	4.0-10.0 x10^3^/µL
Red Blood Cell (RBC)	5.3	4.0-5.2 x10^3^/µL
Hemoglobin	15.1	12.0-16.0 g/dL
Hematocrit	44.6	35.0-45.0 %
Mean Corpuscular Volume (MCV)	83.5	78.0-100.0 fl
Mean Corpuscular Hemoglobin (MCH)	28.3	26.0-34.0 pg
Mean corpuscular hemoglobin concentration (MCHC)	33.9	31.0-36.0 g/dL
Red Cell Distribution Width (RDW)	14.0	11.0-14.6%
Platelet Count	283	150-450x10^3^/µL
Mean Platelet Volume (MPV)	9.5	9.5-12.2 fl
Neutrophil %	84	34-73%
Bands %	9.5	0-10%
Lymphs %	3	25-45%
Monocytes %	3	2-6%
Myelocytes %	0	≤ 0%
Promyelocytes %	0	≤ 0%
Cardiac Markers		
High Sensitivity Troponins Zero Hour	103	<22 ng/L
High Sensitivity Troponins One Hour	113	<22 ng/L
High Sensitivity Troponins Three Hour	123	<22 ng/L
Urinalysis		
Color	Yellow	Amber
Clarity	Hazy	N/A
Specific Gravity	1.016	1.003 - 1.030
pH	6.0	3.5 - 8.0
Protein	Negative	Negative mg/dL
Glucose	> 500	Negative mg/dL
Ketones	Trace	Negative mg/dL
Bilirubin	Negative	Negative mg/dL
Blood	Negative	Negative
Nitrite	Negative	Negative
Urobilinogen	Negative	Normal E.U/dL
Leukocyte Esterase	Negative	Negative
Red Blood Cells	1	0 - 5 HPF
White Blood Cells	1	0 - 5 HPF
Squamous Epithelium	< 1	Not established/HPF
Bacteria	None seen	None seen/HPF

**Figure 1:**
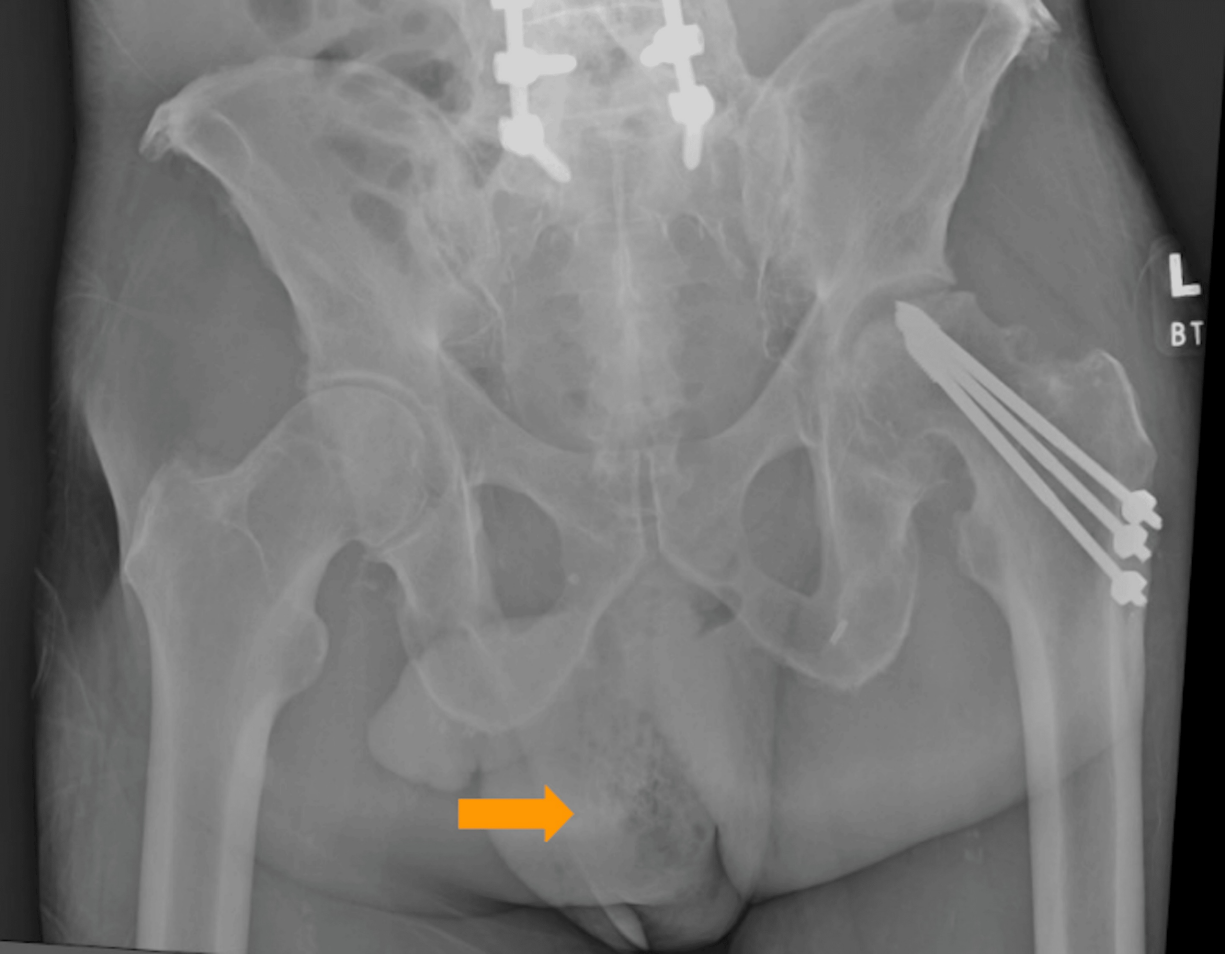
Our patient's x-ray of the left hip revealed subcutaneous gas in the scrotum, concerning gas gangrene (orange arrow) along with progressive severe degenerative changes in the left hip with increased flattening of the femoral head.

**Figure 2:**
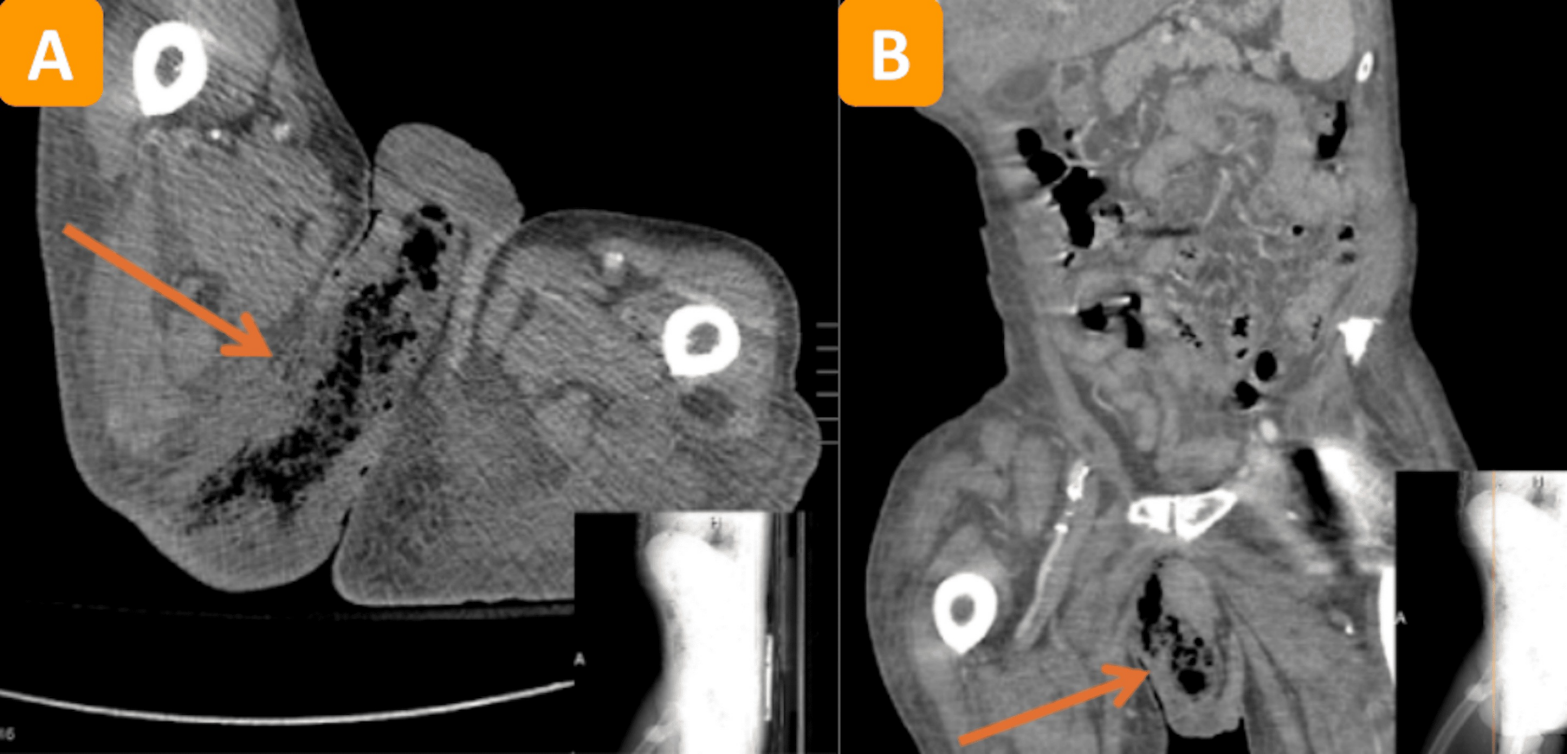
Abdominal and pelvic CT scan The results of our patient's abdominal and pelvic CT scan indicated the presence of necrotizing fasciitis in the right perineum and gluteal region, as noted by the arrows in (A) (axial view) and (B) (coronal view) with the images in the right lower corners demonstrating the location of these specific cross-sectional images.

Imaging included x-rays of the left hip (Figure 1) and femur followed by an abdominal and pelvic computerized tomography (CT) scan (Figures 2A, 2B).

**Figure 3:**
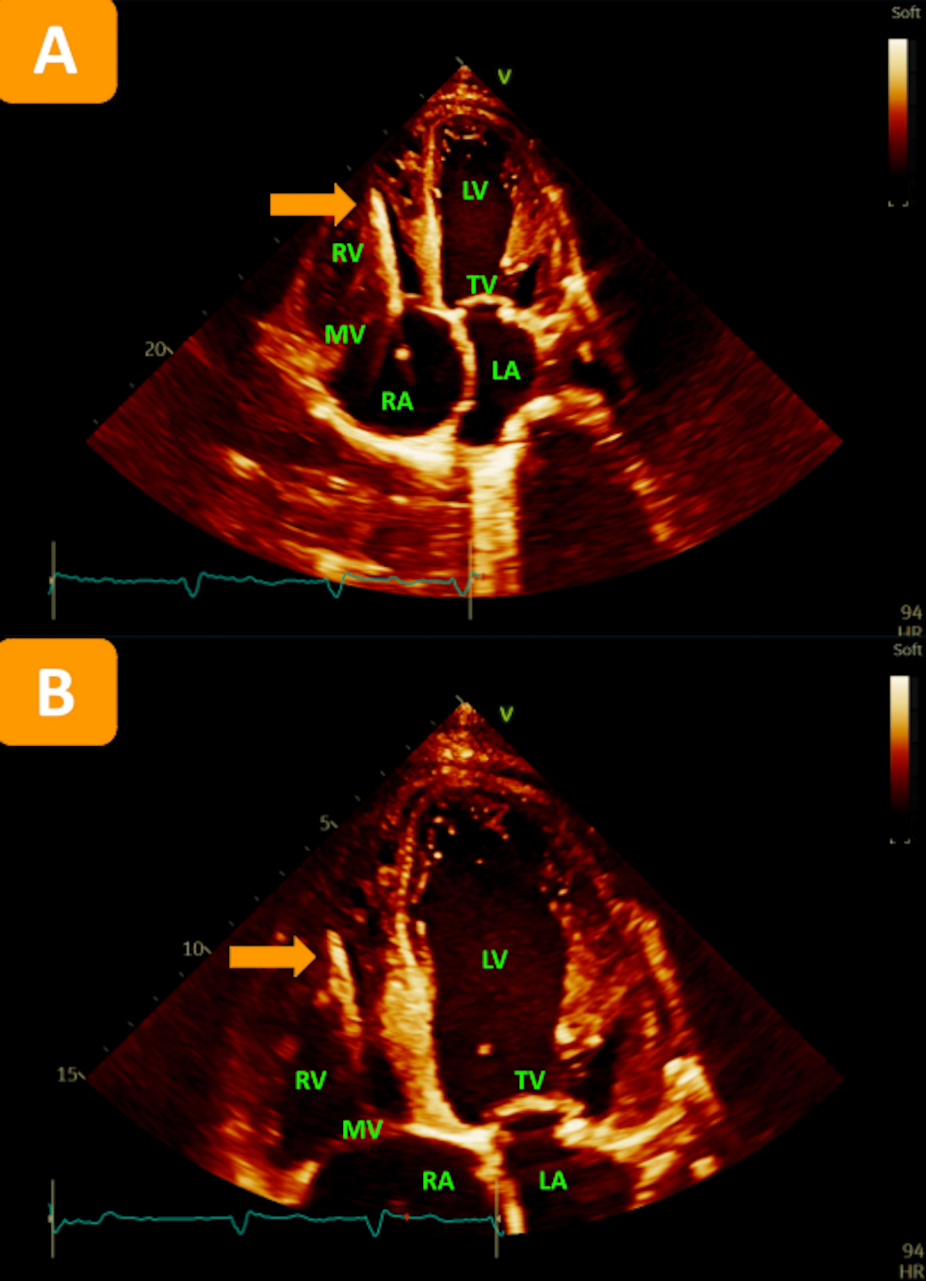
TTE in the apical four-chamber view (A) with a focus on the apex (B) showing no evidence of valvular endocarditis. Left ventricle ejection fraction estimated at 15%-20%. Multiple segments of the LV are hypokinetic. The right ventral is severely dilated and hypokinetic. MV leaflets are sclerotic. Dilated tricuspid valve annulus with tricuspid regurgitation. Echogenic finding suggestive of pacemaker lead noted in right ventricle (orange arrows). TTE: transthoracic echocardiogram, RA: right atrium, RV: right ventricle, MV: mitral valve, LA: left atrium, LV: left ventricle, TV: tricuspid valve.

Initial blood cultures revealed the presence of methicillin-resistant Staphylococcus aureus. Considering this result and suspicion of FG, the patient was promptly put on a regimen of broad-spectrum antibiotics including vancomycin, cefepime, and metronidazole. Additionally, his SGLT2i was discontinued indefinitely. A transthoracic echocardiogram (TTE) ruled out any signs of endocarditis, and subsequent blood cultures came back negative upon multiple repeats (Figures 3A, 3B).

The patient was admitted to the surgical intensive care unit (ICU) with urology following. The surgical team performed a sharp debridement of deep tissues of the perineum, scrotal, and right gluteal regions. Following the procedure, the patient went into undifferentiated shock, thought to be a combination of cardiogenic and septic shock as indicated by invasive hemodynamic monitoring. Vasopressors were initiated, and the patient was intubated for airway protection given his worsening mental status. Throughout the hospitalization, the patient’s condition was further complicated by the development of atrial fibrillation and renal failure, which progressed to requiring continuous renal replacement therapy. Tragically, the patient suffered a cardiac arrest and passed away six weeks after admission.

## Discussion

Between March 1, 2013 and January 31, 2019, the FDA documented 55 instances of FG in individuals prescribed SGLT2is, with the duration from the commencement of SGLT2i therapy to the onset of symptoms varying from five days to forty-nine months [3]. Of the 55 cases, 16 were linked to dapagliflozin specifically [3,8]. Our patient fell within this period, having been prescribed the SGLT2i dapagliflozin two months before presentation. 

In general, identified risk factors linked to the onset of FG include fungal infections, recurrent urinary tract infections, obesity, male gender, advanced age, local trauma, smoking, immunosuppression, poorly managed diabetes, and inadequate personal hygiene, particularly in the genital region [7-9]. The infection typically starts from a local trauma or surgical procedure or spreads from an adjacent infection such as a perianal abscess, urinary tract infection, or skin infection. The bacteria produce toxins and enzymes (such as collagenase and hyaluronidase) that facilitate the rapid spread of the infection and tissue destruction. The condition leads to thrombosis of the small subcutaneous vessels, resulting in tissue ischemia and necrosis that we know as FG. Our patient, an elderly male, exhibited poor hygiene, evidenced by urine saturation due to prolonged immobility, alongside poorly controlled diabetes indicated by a hemoglobin A1c level of 13.5%. The combination of glycosuria and inadequate hygiene during extended immobility likely facilitated bacterial infiltration, leading to the development of FG. 

Identifying FG requires a heightened clinical suspicion because no individual laboratory test or imaging study can conclusively diagnose it, and any delay in diagnosis can lead to increased mortality rates. Treatment requires adequate fluid resuscitation, broad-spectrum antibiotics, and oftentimes aggressive surgical intervention. Delay in surgical intervention is the most substantial modifiable risk factor correlated with mortality in cases of necrotizing soft tissue infections [5]. In the case of our patient, he was promptly started on broad-spectrum antibiotics and taken to the operating room. It should be noted, however, that his history of DVT may have contributed to his mortality, as vascular disease is associated with higher mortality in FG patients [5]. 

Overall, FG is a rare complication of SGLT2is with reports of increased occurrences associated particularly with empagliflozin [10,11]. Dapagliflozin has less commonly been linked to this adverse effect. Moon et al., however, presented a case of FG in a patient on dapagliflozin with well-maintained glycemic levels and no diabetic complications, implying a potential association with the medication [12]. Given the infrequent incidence of FG, further investigation is warranted to ascertain any relationship between SGLT2is and FG [4,10].

## Conclusions

Originally formulated as antidiabetic agents, SGLT2is have gained prominence in medicine for their cardiovascular and renal protective properties. The aim of this case study was to alert physicians to a rare but potentially life-threatening adverse effect of SGLT2is that requires immediate intervention. This warning is crucial because the use of this medicine class extends beyond its original diabetes indications.
